# Effect of Ideal Amino Acid Ratio of Arginine to Lysine on Intake, Nutrient Digestibility, Growth Performance, Antibody Titers of Newcastle Disease and Infectious Bronchitis Disease, and Carcass Characteristics of Broilers

**DOI:** 10.3390/ani15020135

**Published:** 2025-01-08

**Authors:** Wahab Liaqat, Urooj Anwar, Asfa Fatima, Azhar Rafique, Riaz Mustafa, Umar Farooq, Faisal Ramzan, Waseem Abbas, Muhammad Farooq Khalid, Muhammad Ashraf, Muhammad Riaz, Muhammad Qamar Bilal, Muhammad Aziz ur Rahman

**Affiliations:** 1 Institute of Animal and Dairy Sciences, University of Agriculture, Faisalabad 38000, Pakistan; wahabliaqat67@gmail.com (W.L.); uroojziaagri@yahoo.com (U.A.); asfa3396@gmail.com (A.F.); drfaisal.ramzan@uaf.edu.pk (F.R.); waseem.abbas@uaf.edu.pk (W.A.); professorashraf@uaf.edu.pk (M.A.); muhammadriazvirk11107@yahoo.com (M.R.); drqamarbilal@gmail.com (M.Q.B.); 2 Department of Zoology, Government College University, Faisalabad 38000, Pakistan; azharrafique@gcuf.edu.pk; 3 Sub Campus T.T Singh, University of Agriculture Faisalabad, Faisalabad 38000, Pakistan; drriaz@uaf.edu.pk (R.M.); u.farooq@uaf.edu.pk (U.F.); farooq325@uaf.edu.pk (M.F.K.)

**Keywords:** arginine, chicken, ileal digestibility, immune response, nutrition, organ weight

## Abstract

This study aimed to assess the effects of different arginine (Arg) to lysine (Lys) ratios on feed intake, nutrient digestibility, growth performance, carcass characteristics, and antibody titers of Newcastle disease (ND) and infectious bronchitis (IB) disease in broilers during 35 days of trial. For this purpose, a total of 816 day-old broiler birds having an average weight of 38 ± 3 g were divided into six dietary treatments in such a way that each treatment had eight replicates and each replicate had 17 birds. The treatments were 0.95 (1.19/1.25, 1.12/1.18, and 1.05/1.1), 1 (1.25/1.25, 1.18/1.18, and 1.1/1.1), 1.05 (1.31/1.25, 1.24/1.18, and 1.17/1.1), 1.10 (1.38/1.25, 1.30/1.18, and 1.23/1.1), 1.15 (1.44/1.25, 1.36/1.18, and 1.28/1.1), and 1.20 (1.50/1.25, 1.42/1.18, and 1.34/1.1) Arg/Lys divided into different amounts according to the nutritional needs of starter, grower, and finisher diets, respectively. The results showed that in the finisher phase, the FI was influenced cubically, while in the overall period, a quadratic effect was observed for the FI (*p* < 0.05). The results of BWG showed that BWG was linearly increased in birds given different Arg/Lys in the grower, finisher, and overall period (*p* < 0.05). The results also showed that during the grower, finisher, and overall phases, the body weight gain (BWG) was better in birds fed with 1.05 and 1.10 Arg/Lys (*p* < 0.05). Furthermore, a better feed conversion ratio (FCR) was shown in the starter and grower phases at 1.10 and 1.15 Arg/Lys (*p* < 0.05). The results of nutrient digestibility explored showed that the experimental treatments had a better effect (*p* < 0.05) on dry matter and crude fat digestibility in birds fed with 1.19/1.25, 1.12/1.18, and 1.05/1.1 and 1.25/1.25, 1.18/1.18, and 1.1/1.1 Arg/Lys in their diets while the crude protein digestibility remained unaffected with different treatments (*p* > 0.05). The treatments had no effect (*p* > 0.05) on relative organ weights, immune organs, and carcass characteristics. However, higher titers of ND and IBD were observed in birds fed with Arg/Lys of 1.38/1.25, 1.30/1.18, and 1.23/1.1 and 1.50/1.25, 1.42/1.18, and 1.34/1.1 in their diets (*p* < 0.05). Based on the results of the current study, it is concluded that the inclusion levels of 1.38/1.25, 1.30/1.18, and 1.23/1.1, and 1.44/1.25, 1.36/1.18, and 1.28/1.1 Arg/Lys resulted in better growth performance and immune response in the broiler birds, respectively. Therefore, adjusting the levels of Arg/Lys in feed is recommended to enhance broilers’ growth performance and immune response.

## Simple Summary

The ideal arginine/lysine is maintained in broiler ration to reach the target weight without wasting nitrogen content in the environment. An improved body weight gain at 1.05 and 1.10 arginine/lysine and a better feed conversion ratio at 1.10 and 1.15 arginine/lysine were shown during the current trial. The nutrient digestibility of dry matter and crude fat was improved, while the digestibility of crude protein remained unaffected by the arginine/lysine in the diet. Moreover, better titers of Newcastle disease and infectious bursal disease were observed at a higher Arg/Lys. Thus, this study provides evidence that adjusting the arginine/lysine ratio in feed is recommended to enhance broilers’ growth performance and immune response.

## 1. Introduction

Amino acids are building blocks of protein and regulate different pathways that are necessary for growth, maintenance, metabolism, and immunity in birds [1]. Arginine (Arg) is considered an essential amino acid for poultry [2] because broilers are unable to synthesize Arg due to a lack of major key enzymes like ornithine carbamoyl-transferase, hepatic Arg, and carbamoyl phosphate synthase I in the urea cycle [3]. Ball et al. [1] reported that an Arg requirement is higher among broilers than other birds because of the low synthesis of endogenous Arg and protein deposition for rapid growth. Arginine plays an important role in protein synthesis, growth, immunity, and some metabolic pathways and acts as a precursor for different components, including nitric oxide, proline, and glutamate [1,4]. A study by Al-Daraji and Salih [5] indicated that adding Arg to the diet at a level of 0.06% resulted in increased growth performance and a better feed conversion ratio (FCR) of the broilers. Arginine also stimulates the release of insulin, insulin-like growth factor, and growth hormone in the bloodstream [6] and acts as a precursor of nitric oxide. Nitric oxide plays a role as a potent vasodilator and an important component of macrophages in birds, which are involved in phagocytosis and thus improve immunity [4]. Nitric oxide also lowers tension by relaxing the smooth muscle of vessels and secreting different vasoconstrictors like endothelin-1 and serotonin [7].

Lysine (Lys) is another essential amino acid, considered one of the limiting amino acids in poultry, and is required for the optimum production of breast meat and live performance traits in broilers [8]. Lysine had a significant effect on body weight gain (BWG), FCR, and carcass characteristics [9]. However, any excess or deficiency of the arginine to lysine ratio (Arg/Lys) could have negative effects on growth performance by altering the concentrations of amino acids in muscles [10]. Balnave and Brake [10] reported that this adverse effect is more evident with high Lys (low Arg: Lys) than a diet with high Arg (high Arg/Lys). As demonstrated by Sigolo et al. [11], 110% and 120% (of Ross recommendations) Lys levels reduced the average daily feed intake (FI), ultimately resulting in poor BWG. Ale Saheb Fosoul et al. [12] also concluded that birds fed an Arg-deficient diet had a lower body weight (BW) and poor FCR than birds fed an Arg diet. However, supplementing 0.09% Arg not only increased BWG but also resulted in better FCR, supporting that an optimal level of Arg is required for the overall performance of broilers [13].

Based on the previous studies, it could be hypothesized that Arg and Lys are two important amino acids in a broiler diet that impact the performance parameters of birds. Therefore, an Arg/Lys ratio in the diet of the broiler should be optimized to achieve better performance. This study aimed to assess the effects of supplementation of the ideal Arg/Lys on the immune response, growth performance, and carcass characteristics in broilers.

## 2. Materials and Methods

### 2.1. Birds and Management

The current study was carried out at Paroka Poultry Research Center, University of Agriculture, Faisalabad, Pakistan. The handling and slaughtering procedure of the broiler chickens was approved by the Advanced Studies and Research Board, University of Agriculture, Faisalabad (IADS/2020/1667).

In the current study, a total of 816 day-old Ross broiler chicks were divided into 6 dietary treatments, and each treatment contained 8 replicates with 17 birds in each replicate. The size of the pen (replicate) was 2.43 m × 0.91 m × 1.21 m (length × width × height) to provide maximum space for the birds to access feed and water freely. On day one, all the chicks were weighed, and the average weight of the chicks was noted as 38 ± 3 g. The bedding material used in the rearing of birds consisted of rice hulls in a 2-inch layer. The experimental shed temperature was maintained at 35 °C one day before the arrival of the chicks. Birds were reared at a 35 °C temperature for the first week and the temperature was reduced weekly by 2.78 °C until reaching 24 °C, following the protocol as suggested by Ketelaars [14]. A photoperiod of 23 h was provided from 0 to 7 days and the last 3 days of the trial, while light for 18 h was provided during the remaining period of the trial according to the standards of the European Union [15]. Ad libitum feed and water were offered to the chicks throughout the experiment.

### 2.2. Experimental Design and Diets

The duration of the experiment was 35 days. Three diets, which were the starter (0–7 days), grower (8 to 21 days), and finisher (22 to 35 days), were prepared. The experimental treatments consisted of six dietary treatments, which were 0.95 (1.19/1.25, 1.12/1.18, and 1.05/1.1), 1 (1.25/1.25, 1.18/1.18, and 1.1/1.1), 1.05(1.31/1.25, 1.24/1.18, and 1.17/1.1), 1.10 (1.38/1.25, 1.30/1.18, and 1.23/1.1), 1.15 (1.44/1.25, 1.36/1.18, and 1.28/1.1), and 1.20 (1.50/1.25, 1.42/1.18, and 1.34/1.1) Arg/Lys for the starter, grower, and finisher diets, respectively, to evaluate the varying Arg/Lys levels at each phase.

The ingredients used for the formulation of the experimental diets were chemically analyzed for dry matter (DM), crude protein (CP), and crude fat (CF) before feed formulation by the AOAC (2004) methods, as demonstrated by Onuoha et al. [16]. The nutrient requirements of the birds were kept at the standards given by NRC [17] during the starter, grower, and finisher diets. The inclusion level of the ingredients in the diet is presented in Table 1, while the chemical composition of the experimental rations is presented in Table 2.

### 2.3. Feed Intake and Growth Performance

Feed intake and growth performance parameters like BWG and FCR were calculated every week, as described in previous studies [18,19]. In brief, the FI was measured by a difference method in which the difference between the offered feed and refusal was calculated. To calculate the weekly FI per bird, the total feed intake per replicate was divided by the total number of birds per replicate. Mortality of the birds in each replicate was observed daily to calculate the corrected FI and to avoid any chances of error. Similar to the FI, the BWG of the birds was also determined on a weekly basis. For this purpose, the initial body weight of the chicks was determined on the day of arrival, and after that, birds in each replicate were weighed on a weekly basis. To determine the BWG weekly, the difference method was used as mentioned for the FI measurements. Hence, the weekly BWG was calculated by the difference between the initial body weight of the birds at the start of the week and the final BW at the end of the week. To determine the FCR, the feed consumed per bird in a given duration was divided by the weight gain during the said duration.

### 2.4. Nutrient Digestibility

On the 35th day of the experimental trial, the indirect marker (acid-insoluble ash, AIA) procedure was used to find out the digestibility of the nutrient. Celite^®^ (Celite Corp., Lompoc, CA, USA), an AIA, was added to the experimental diets of the broilers that continued until the 35th day of the trial. To determine the ileal nutrient digestibility, four birds per pen were randomly selected and slaughtered through cervical dislocation. The ileum was demarcated as the portion of the small intestine extending from Meckel’s diverticulum to the 40 mm cranial to an ileocecal junction. The ileal contents were flushed in a 200 mL plastic cup, with a few drops of formalin added to stop any microorganism activity. The plastic cups were immediately transferred into an ice container to freeze them at −10 °C, as demonstrated by Khalil et al. [20]. The collected fecal samples were transferred to the lab for further analysis.

### 2.5. Carcass Characteristics, Immune Organ Weight, and Antibody Titers

On the last day of the experimental trial, one bird from each replicate was selected randomly and slaughtered to calculate the different carcass characteristics, such as the live weight of the bird, dressing percentage, breast muscle, thigh meat percentage, and the relative weight of the gizzard, liver, and heart, as calculated in a previous studies by Ramaiyulis et al. [21] and Ishaq et al. [22]. On slaughtering, the weight of different immune organs like the thymus, spleen, and bursa were also recorded to check the relative organ weight % by using the formula such as the weight of the organ divided by the carcass weight multiplied by 100. Antibody titers for Newcastle disease (ND) virus were measured using the heme-agglutination inhibition technique used by Ishaq et al. [22] and Allan and Gough [23]. Serum collected for infectious bronchitis virus (IBV) titers were measured by a commercial ELISA kit, as demonstrated by Boudaoud et al. [24].

### 2.6. Chemical Analysis

For the chemical analysis of the feed and feces, samples were collected from each replicate and combined to make eight composite samples separately for each treatment. The samples were then dried at 65 °C using the hot air oven (Heraeus, Hanau, Germany), desiccated, and powdered to easily pass through a sieve of 0.5 mm. The grounded samples were preserved at a −20 °C temperature until further analysis for DM, CP, and CF following the standard methodologies described in past investigations [20]. The Kjeldahl method was used to measure the nitrogen content of the sample to further calculate the CP by multiplying the nitrogen concentration by 6.25. Furthermore, the Soxhlet apparatus was utilized for the determination of crude fat in the feed sample as well as feces. Acid-insoluble ash was also measured using the ash samples of the feed and feces, as well.

### 2.7. Calculations

To determine the apparent nutrient digestibility coefficient, the following formula was used:

$$\mathrm{Digestibility}\;\mathrm{coefficient}\;(\%)=100-(100\times \frac{\%\;\mathrm{marker}\;\mathrm{in}\;\mathrm{feed}}{\%\;\mathrm{marker}\;\mathrm{in}\;\mathrm{feces}}\times \frac{\%\;\mathrm{nutrient}\;\mathrm{in}\;\mathrm{feces}}{\%\;\mathrm{nutrient}\;\mathrm{in}\;\mathrm{feed}})$$

### 2.8. Statistical Analysis

Before performing the statistical analysis, the mortality percentages were transformed using arcsine transformation. Collected data were then evaluated using one-way ANOVA under the Completely Randomized Design of the statistical software Minitab 17 [25]. Tukey’s post hoc test was used to determine the significant differences between the treatment groups. Polynomial contrasts were also performed. The differences with *p*-values less than 0.05 were considered statistically different.

## 3. Results

### 3.1. Growth Performance

The data regarding growth performance are provided in Table 3. The results of the growth performance indicated that different Arg/Lys ratios in starter and grower diets did not affect the FI (*p* > 0.05). However, a considerable difference in the FI was observed in birds given different Arg/Lys diets during the finisher phase and overall period (*p* < 0.05). In the finisher phase, the FI was influenced cubically, while in the overall period, a quadratic effect was observed for the FI (*p* < 0.05). The results of BWG showed that the BWG was linearly increased in birds given different Arg/Lys diets in the grower, finisher, and overall period (*p* < 0.05). Moreover, an analysis of the collected data indicated that the FCR decreased linearly (*p* < 0.05) in birds given different Arg/Lys ratios in the grower phase, while the FCR was influenced quadratically in the starter phase (*p* < 0.05). The birds that received 1.05, 1.10, and 1.15 levels showed a better FCR (*p* < 0.05). However, no significant difference in the FCR was shown during the overall period (*p* > 0.05).

### 3.2. Digestibility

The results of nutrient digestibility, including DM digestibility, CP digestibility, and crude fat digestibility, are shown in Table 4. The statistical analysis of the data showed a better effect on DM digestibility in birds fed different levels of Arg/Lys (*p* < 0.05). The experimental treatments had a linear effect on DM digestibility (*p* < 0.05). The highest DM digestibility was observed in birds that received 0.95 and 1.00, and the lowest value was observed in birds fed with 1.15. On the contrary, no difference (*p* > 0.05) in CP digestibility was observed in birds given different Arg/Lys diets. However, a linear difference in CF digestibility was observed in birds given different Arg/Lys (*p* < 0.05) diets, and higher CF digestibility was observed in birds fed with the 0.95 and 1.00 Arg/Lys diets (*p* < 0.05).

### 3.3. Carcass Characteristics, Immune Organs Weights, and Antibody Titers

The results of the carcass characteristics, including the percentages of dressing, breast, thigh, heart, gizzard, and liver, are shown in Table 5. The results showed that different Arg/Lys diets did not affect carcass parameters (*p* > 0.05). In the current study, no effect on relative bursa, spleen, and thymus weight was observed in the different dietary levels of Arg/Lys (*p* > 0.05) (Table 6). However, a quadratic effect in the log value of the ND virus in broilers was observed for the experimental treatments 1.10 and 1.20 Arg/Lys (*p* < 0.05). Similarly, the statistical data analysis indicated a linear impact on the IBV titers in birds given different Arg/Lys diets (*p* < 0.05). The birds that were fed with Arg/Lys of 1.10 and 1.20 had the highest titers against IBV than birds fed with other treatments.

## 4. Discussion

Arginine not only acts as a building block for protein but also plays a major role as a precursor for substances that affect different biological processes [26]. This renders Arg in a key position in several physiological functions, ensuring its adequate presence in broiler nutrition [26,27]. Several studies have assessed the impact of Arg supplementation on FI, growth performance, carcass characteristics, and antibody titers in broilers with very high levels of arginine [28,29]. For example, in the study by Ebrahimi [30], a high Arg level in broiler feed was used up to 24 g/kg without modifying the lysine concentration. The current study was planned to check the impact of the range of Arg/Lys (0.95 to 1.20) supplementation according to the physiological stages of broilers on their FI, growth performance, carcass characteristics, and immune response. In the current study, the FI was not influenced by different Arg/Lys ratios in starter and grower diets. This is similar to the findings of Murakami et al. [30], who studied the impact of Arg supplementation in broiler breeders and evaluated no significant effect on the FI, with levels of Arg (1.39% to 1.79%) corresponding to Arg/Lys ranging from 1.103 to 1.421 being present in the feed during the starter phase. Similarly, Ebrahimi et al. [29] studied the effect of excess arginine on the growth performance of broilers and reported that 1.18 and 1.20 Arg/Lys resulted in no significant difference in the FI of growing broilers. The unaffected FI indicates that weight gain fluctuated mainly because of the efficient feed utilization by birds and adequate availability of amino acids in the diet.

Arginine releases different growth hormones, such as insulin-like growth factor, glucagon, and growth hormone [28], which could enhance the BWG in broilers. In the current study, the 1.05 and 1.10 Arg ratios increased the BWG in broilers during the grower, finisher, and overall phase broilers, which could be attributed to higher DM and CF digestibility and due to Arg’s bio-availability for protein synthesis as well as the release of different hormones such as glucagon and growth hormones [5]. But, in the starter phase, the Arg/Lys did not influence the BWG in broilers, which is similar to the findings of Murakami et al. [30], who studied the impacts of Arg supplementation during the starter phase on broiler production performance, having a corresponding Arg/Lys ranging from 1.103 to 1.421, and observed no significant effect on BWG by Arg supplementation. Similarly, the impact of excess arginine supplementation on lipogenic gene expression and growth performance in broilers was examined by Ebrahimi et al. [29], having 1.18 and 1.20 Arg/Lys in the feed; they also noticed an unaffected BWG in broilers. Interestingly, the impact of supplementation of guanidinoacetic acid and arginine in broiler birds was studied by Esser et al. [31] using 1.29, 1.15, and 1.08 Arg during the starter, grower, and finisher phases, respectively, having a variable Arg/Lys (1.05 to 1.08), and observed no impact on BWG during the finisher and overall phases. No impact on BWG due to different levels of Arg/Lys in the diet of broilers may be due to the different interrelationships between the dietary amino acids that affect the amount of catabolism and amino acids interaction that masked the effects [31,32]. Furthermore, polyamines, which are generated from ornithine, are the precursor of Arg, as stated by Castro et al. [33], resulting in improved FCR. In the current study, improved FCRs in the 1,05, 1.10, and 1.15 treatments could be attributed to the higher availability of ornithine that is generated after Arg metabolism. It has also been reported that Arg is a precursor of polyamines, such as spermine and spermidine, that act on the intestinal epithelium, resulting in the maturation of intestinal epithelium and villi that result in a better absorption of nutrients and better feed efficiency [34]. Therefore, a high level of Arg during the starter and grower phases resulted in better maturation of the intestinal epithelium, which resulted in better feed efficiency in birds.

Regarding the dressing percentage, no significant difference in the dressing percentage and thigh and breast muscle yield was observed in the current study, which is similar to the findings of the studies by Mejia et al. [35], who used 1.15, 1.0, and 1.30 Arg/Lys from 28 to 42 days in the broiler diet and reported that no effect on the breast meat yield with increasing Arg/Lys was observed. Similarly, Cengiz and Kucukersan [36] used graded levels of arginine in the diet for broilers, where the Arg ratio was added with corresponding 1.04 and 1.07 Arg/Lys during the starter and grower phases, respectively, and found that these Arg/Lys ratios have no impact on the dressing percentage. There is a possibility that the environmental conditions under which broilers were reared during the trial were not optimal for the supplemented Arg/Lys to have an impact. Notably, in the current study, eight samples per treatment for the carcass characteristics analysis is a limitation that may affect the statistical results of the experiment. Therefore, for the validity of the results, a large sample size per treatment is essential for further studies. Similarly, relative organ weights in birds fed with 0.95 to 1.20 Arg ratios were similar, and the experimental treatment had no effect on relative organ weights, which is similar to the study by Yang et al. [37], who studied the impacts of arginine supplementation with 0, 8.5, and 17 mg of L-arginine/kg during 42 days in laying hens and reported that Arg supplementation did not influence the relative organ weights in laying hens. The bursa and thymus are fundamental lymphoid organs and are the regions of maturation for B and T lymphocytes. Lymphoid organs are essential for the post-hatching development of birds and slowly reduce in size and deprive functionality as the bird matures [38]. Unlike the results of the current study, Jahanian [39] noticed a heavily weighted thymus when a diet during the starter phase was fed with 80, 90, 100, 110, and 120% of the Arg levels of the NRC recommendations, pointing out that lymphoid organs’ development may only be compromised when Arg is insufficient in the broiler diet. Interestingly, a remarkable effect on antibody titers of ND in birds with a 1.10 Arg ratio was observed, which was according to the study by Kidd et al. [40]. They studied the impact of dietary Arg on the growth and immunity of broiler chicks having 1.27 and 1.13 Arg/Lys during the trial, which resulted in a remarkable effect on the antibody titer of ND. Similarly, immunological responses in the starting phase of broilers by supplementation of dietary protein and arginine were studied by Jahanian [39], who supplemented Arg in diets having 1.15 and 0.90 Arg/Lys and observed higher antibody titers of ND virus by supplementation. Arginine likely acts as a precursor for nitric oxide production, a major component of avian macrophage, which results in phagocytosis and improves the number of lymphocytes. These resulted in the modulation of the humoral response due to an increased number of cells [41]. The results were in contrast to the study results by Bulbul et al. [42], who studied the impact of dietary L-arginine on antibody titers having 90–130% Arg levels of the NRC recommendations with 1.05 and 0.97 Arg/Lys in the diet, they noticed no effect on the ND titers in broiler birds. These findings suggest that dietary L-Arg supplementation does not affect the symptoms after vaccination, especially at the starter and finisher phases.

Similarly, the birds fed with 1.10 Arg in the diet showed a positive impact on antibody titers of the IB virus. Similar findings have also been reported by Jahanian and Khalifeh-Gholi [43], who studied the impacts of arginine and methionine on immunological responses in broiler chickens having 1.02 and 0.99 Arg/Lys in the diet during the starter and grower phases, respectively, and concluded that an increased antibody titer against IBV resulted in broilers. An increased antibody response against IBV might be because of the arginine pathway through which polyamines are produced, involved in the mitogenesis of lymphocytes [27], and the nitric oxide pathway during which arginine is transformed into nitric oxide. Nitric oxide has important roles in the coagulation and maintenance of the immune system [44,45].

## 5. Conclusions

This study highlights that adjusting the Arg/Lys in broiler diets enhances growth performance, feed conversion, and immune responses. The ratios 1.38/1.25 and 1.44/1.25 improved the feed conversion, body weight gain, and antibody titers against Newcastle disease and infectious bronchitis. While no significant effects were observed for the carcass traits or organ weights, these findings suggest that optimizing Arg/Lys can effectively boost broiler productivity and immunity. Further studies should be conducted to evaluate the persistent impacts of Arg/Lys on gut microbiota and disease resistance in poultry so that an optimal potential of adjusted Arg/Lys can be achieved.

## Figures and Tables

**Table 1:** Ingredient composition (%) of diets during starter, grower, and finisher phases offered with varying concentrations of arginine/lysine (as-fed basis).

	Starter Diet (0–7 Days)						Grower Diet (7–21 Days)						Finisher Diet (22–35 Days)					
Ingredients	Arginine/Lysine						Arginine/Lysine						Arginine/Lysine					
	0.95	1.00	1.05	1.10	1.15	1.20	0.95	1.00	1.05	1.10	1.15	1.20	0.95	1.00	1.05	1.10	1.15	1.20
Maize 12%	59.22	56.71	54.21	51.71	51.13	51.00	61.65	59.28	56.92	55.35	55.23	55.10	62.09	59.82	57.54	56.56	65.45	56.33
^1^ CG 60%	1	1	1	1	1	1	1	1	1	1	1	1	1	1	1	1	1	1
^2^ SBM	20.93	23.24	25.54	27.84	28.29	28.31	16.51	18.69	20.86	22.27	22.29	22.32	14.93	17.01	19.09	19.94	19.96	19.98
^3^ RSM 36%	2	2	2	2	2	2	2	2	2	2	2	2	2	2	2	2	2	2
Canola meal	10	10	10	10	10	10	10	10	10	10	10	10	10	10	10	10	10	10
Poultry oil	0.31	0.75	1.19	1.64	1.75	1.8	0.97	1.39	1.81	2.09	2.13	2.17	2.38	2.78	3.18	3.36	3.4	3.43
^4^ PBM	2	2	2	2	2	2	4	4	4	4	4	4	4	4	4	4	4	4
L-Arginine	-	-	-	-	0.05	0.11	-	-	-	0.02	0.08	0.14	-	-	-	0.03	0.09	0.15
^5^ DCP	0.95	0.93	0.9	0.87	0.87	0.87	0.57	0.55	0.52	0.51	0.51	0.51	0.26	0.24	0.22	0.21	0.21	0.21
Marble chips	0.57	0.57	0.57	0.56	0.56	0.56	0.43	0.42	0.42	0.42	0.42	0.42	0.48	0.47	0.47	0.47	0.47	0.47
Salt (NaCl)	0.25	0.25	0.25	0.25	0.25	0.25	0.19	0.19	0.19	0.19	0.19	0.19	0.19	0.19	0.19	0.19	0.19	0.19
^6^ Na_2_HCO_3_	0.1	0.1	0.1	0.1	0.1	0.1	0.1	0.1	0.1	0.1	0.1	0.1	0.1	0.1	0.1	0.1	0.1	0.1
Lysine sulphate	0.63	0.53	0.44	0.35	0.33	0.33	0.63	0.54	0.45	0.39	0.39	0.39	0.61	0.52	0.44	0.4	0.4	0.4
L-isoleucine	0.12	0.08	0.05	0.01	-	-	0.12	0.08	0.05	0.03	0.03	0.03	0.12	0.09	0.06	0.04	0.04	0.04
L-threonine	0.12	0.09	0.06	0.03	0.03	0.03	0.1	0.08	0.05	0.03	0.03	0.03	0.11	0.08	0.06	0.05	0.05	0.05
DL-Methionine	0.2	0.18	0.17	0.15	0.15	0.15	0.15	0.14	0.12	0.11	0.11	0.11	0.18	0.17	0.16	0.16	0.16	0.16
L Valine	0.11	0.08	0.04	0.01	-	-	0.09	0.05	0.02	-	-	-	0.08	0.04	0.01	-	-	-
Choline Cl	0.05	0.05	0.05	0.05	0.05	0.05	0.05	0.05	0.05	0.05	0.05	0.05	0.05	0.05	0.05	0.05	0.05	0.05
Betaine	0.07	0.07	0.07	0.07	0.07	0.07	0.07	0.07	0.07	0.07	0.07	0.07	0.07	0.07	0.07	0.07	0.07	0.07
Phytase premix	0.5	0.5	0.5	0.5	0.5	0.5	0.5	0.5	0.5	0.5	0.5	0.5	0.5	0.5	0.5	0.5	0.5	0.5
^7^ Premix	0.87	0.87	0.87	0.87	0.87	0.87	0.87	0.87	0.87	0.87	0.87	0.87	0.87	0.87	0.87	0.87	0.87	0.87
Total	100	100	100	100	100	100	100	100	100	100	100	100	100	100	100	100	100	100

^1^ CG 60%: corn gluten meal 60%, ^2^ SBM: soybean meal, ^3^ RSM: rapeseed meal, ^4^ PBM: poultry by-product meal, ^5^ DCP inorganic: Di-calcium phosphate inorganic, ^6^ Na_2_HCO_3_: sodium bicarbonate, ^7^ Premix: vitamin A 5,600,000 IU, cholecalciferol (Vit. D3) 1,760,000 IU, vitamin E 16,800 IU, riboflavin 3.2 g, d-calcium pantothenate 6.4 g, vitamin B12 7.2 mg, 36 g niacin 0.7 g folic acid, 80 mg biotin, and 2.0 g pyridoxine. Mineral premix: Cu 66 mg; 88 mg Mn; Zn 88 mg; 8.5 mg Fe, and 0.30 mg/kg Se.

**Table 2:** Chemical composition of diets (starter, grower, and finisher) offered with varying concentrations of arginine/lysine (as-fed basis).

	Starter Diet (0–7 Days)						Grower Diet (7–21 Days)						Finisher Diet (22–35 Days)					
Ingredients	Arginine/Lysine						Arginine/Lysine						Arginine/Lysine					
	0.95	1.00	1.05	1.10	1.15	1.20	0.95	1.00	1.05	1.10	1.15	1.20	0.95	1.00	1.05	1.10	1.15	1.20
^1^ CP (%)	21.82	22.52	23.22	23.93	24.06	24.06	21.42	21.81	22.47	22.90	22.90	22.90	19.80	20.43	21.09	21.40	21.52	21.63
Moisture (%)	11.98	11.81	11.61	11.43	11.38	11.37	11.91	11.74	11.57	11.45	11.44	11.43	11.80	11.93	11.88	11.90	11.82	11.85
^2^ ME (kcal/kg)	2850	2850	2850	2850	2850	2850	2950	2950	2950	2950	2950	2950	3050	3050	3050	3050	3050	3050
^3^ DM (%)	88.02	88.21	88.39	88.57	88.62	88.63	88.09	88.26	88.43	88.55	88.56	88.57	88.20	88.07	88.12	88.10	88.18	88.15
^4^ CF (%)	2.59	2.65	2.71	2.77	2.78	2.77	2.42	2.48	2.53	2.57	2.57	2.57	2.25	2.31	2.37	2.43	2.43	2.43
^5^ EE (%)	3.81	4.21	4.61	5.01	5.12	5.16	4.91	5.29	5.67	5.93	5.96	6.00	5.91	6.31	6.71	7.10	7.23	7.26
Ash (%)	3.16	3.28	3.4	3.53	3.55	3.55	3.05	3.17	3.28	3.36	3.36	3.36	2.94	3.06	3.18	3.31	3.34	3.34
Calcium (total)	0.9	0.9	0.9	0.9	0.9	0.9	0.8	0.8	0.8	0.8	0.8	0.8	0.7	0.7	0.7	0.7	0.7	0.7
^6^ Dig. Calcium	0.3	0.3	0.3	0.3	0.3	0.3	0.3	0.3	0.3	0.3	0.3	0.3	0.3	0.3	0.3	0.3	0.3	0.3
^7^ Dig. Phosphorus	0.72	0.72	0.72	0.73	0.73	0.73	0.65	0.65	0.65	0.66	0.66	0.66	0.26	0.26	0.26	0.26	0.26	0.26
Sodium	0.36	0.35	0.34	0.34	0.34	0.34	0.3	0.29	0.29	0.28	0.28	0.28	0.13	0.13	0.13	0.13	0.13	0.13
Threonine	0.79	0.79	0.79	0.79	0.79	0.79	0.76	0.76	0.76	0.76	0.76	0.76	0.73	0.73	0.73	0.73	0.73	0.73
Methionine + Cysteine	0.88	0.88	0.88	0.87	0.88	0.88	0.83	0.83	0.83	0.83	0.83	0.83	0.78	0.78	0.78	0.78	0.78	0.78
Methionine	0.14	0.14	0.14	0.14	0.14	0.14	0.25	0.25	0.25	0.25	0.25	0.25	0.39	0.40	0.42	0.42	0.42	0.42
Arginine	1.19	1.25	1.31	1.38	1.44	1.5	1.12	1.18	1.24	1.3	1.36	1.42	1.05	1.11	1.17	1.23	1.28	1.34
Lysine	1.25	1.25	1.25	1.25	1.25	1.25	1.18	1.18	1.18	1.18	1.18	1.18	1.11	1.11	1.11	1.11	1.11	1.11
Tryptophan	0.21	0.22	0.23	0.24	0.25	0.25	0.19	0.21	0.22	0.22	0.22	0.22	0.39	0.40	0.42	0.42	0.42	0.42
Arginine: Lysine	0.95	1	1.05	1.1	1.15	1.2	0.95	1	1.05	1.11	1.15	1.20	0.95	1.00	1.05	1.10	1.15	1.20

^1^ CP: crude protein, ^2^ ME: metabolizable energy, ^3^ DM: dry matter, ^4^ CF: crude fiber, ^5^ EE: ether extract, ^6^ Dig. Calcium: digestible calcium, ^7^ Dig. Phosphorus: digestible phosphorus.

**Table 3:** Impact of different arginine/lysine diets on the growth performance of the broilers.

Treatments Arginine/Lysine									Significance		
Parameters	0.95	1.00	1.05	1.10	1.15	1.20	SEM	*p*-Value	Linear	Quadratic	Cubic
Starter Phase (0–7 days)											
^1^ FI	103.6	94.0	106.6	91.3	102.4	94.0	5.93	0.361	0.342	0.430	0.421
^2^ BWG	107.1	114.9	123.4	118.5	119.1	112.5	5.33	0.380	0.460	0.325	0.390
^3^ FCR	0.960 ^a^	0.817 ^ab^	0.861 ^ab^	0.706 ^b^	0.861 ^ab^	0.831 ^ab^	0.04	0.025	0.210	0.0362	0.123
Grower Phase (7–21 days)											
FI	723.9	827.0	844.2	854.8	794.4	785.2	34.90	0.141	0.121	0.179	0.213
BWG	563.0 ^b^	664.7 ^ab^	695.3 ^a^	706.7 ^a^	666.7 ^ab^	653.9 ^ab^	27.31	0.010	0.009	0.021	0.129
FCR	1.289 ^a^	1.244 ^ab^	1.213 ^b^	1.208 ^b^	1.190 ^b^	1.199 ^b^	0.01	0.012	0.011	0.092	0.510
Finisher Phase (21–35)											
FI	1707.6 ^b^	1808.2 ^ab^	1818.3 ^ab^	1855.2 ^a^	1815.0 ^ab^	1727.0 ^b^	34.31	0.035	0.121	0.342	0.021
BWG	1184.3 ^b^	1266.2 ^ab^	1329.7 ^a^	1294.7 ^b^	1298.4 ^ab^	1272.5 ^ab^	25.60	0.018	0.013	0.025	0.341
FCR	1.446	1.432	1.368	1.433	1.399	1.365	0.03	0.491	0.345	0.154	0.462
Overall Period (0–35 days)											
FI	2535.1 ^b^	2729.2 ^a^	2769.2 ^a^	2801.3 ^a^	2711.9 ^a^	2606.3 ^ab^	62.72	0.045	0.324	0.043	0.012
BWG	1854.4 ^b^	2045.9 ^a^	2148.5 ^a^	2119.9 ^a^	2084.4 ^a^	2039.0 ^a^	40.21	0.031	0.043	0.027	0.346
FCR	1.367	1.335	1.288	1.321	1.300	1.279	0.02	0.109	0.123	0.254	0.541

^1^ FI: feed intake, ^2^ BWG: body weight gain, ^3^ FCR: feed conversion ratio, SEM: standard error of means based on 8 replicates; ^a,b^ means within a row with (^a^, ^ab^, ^b^) superscripts differ significantly (*p* < 0.05).

**Table 4:** Impact of different arginine/lysine diets on the digestibility percentage of dry matter, crude protein, and crude fat in broilers.

Treatments Arginine/Lysine									Significance		
Digestibility %	0.95	1.00	1.05	1.10	1.15	1.20	SEM	*p*-Value	Linear	Quadratic	Cubic
^1^ DM	88.7 ^a^	90.2 ^a^	87.4 ^ab^	85.7 ^ab^	79.5 ^b^	87.7 ^ab^	1.99	0.028	0.012	0.015	0.281
^2^ CP	55.3	58.4	51.0	41.8	48.9	53.8	4.15	0.105	0.213	0.443	0.345
^3^ CF	84.9 ^a^	85.2 ^a^	82.9 ^ab^	80.2 ^ab^	73.1 ^b^	81.2 ^ab^	1.89	0.019	0.012	0.045	0.327

^1^ DM: dry matter, ^2^ CP: crude protein, ^3^ CF: crude fat, SEM: standard error of means based on 8 replicates; ^a,b^ means within a row with (^a^, ^ab^, ^b^) superscripts differ significantly (*p* < 0.05).

**Table 5:** Impact of different arginine/lysine diets on the carcass characteristics in broilers.

Treatments Arginine/Lysine									Significance		
Parameters %	0.95	1.00	1.05	1.10	1.15	1.20	SEM	*p*-Value	Linear	Quadratic	Cubic
Dressing	61.87	63.55	63.38	64.39	62.28	61.6	1.22	0.560	0.453	0.654	0.379
Breast	43.77	44.33	44.34	44.35	43.77	42.84	0.75	0.683	0.798	0.540	0.471
Thigh	28.34	28.53	29.43	29.59	28.85	29.13	0.49	0.450	0.650	0.390	0.589
Heart	0.58	0.54	0.55	0.52	0.63	0.62	0.04	0.427	0.734	0.322	0.435
Gizzard	1.67	1.63	1.49	1.43	1.58	1.52	0.08	0.399	0.549	0.368	0.553
Liver	2.76	2.35	2.39	2.22	2.48	2.41	0.15	0.331	0.410	0.566	0.398

SEM: standard error of means based on 8 replicates.

**Table 6:** Impact of different arginine/lysine on the relative weight of immune organs (% of body weight), antibody titer of Newcastle disease and infectious bronchitis in broilers.

Treatments Arginine/Lysine										Significance	
Parameters %	0.95	1.00	1.05	1.10	1.15	1.20	SEM	*p*-Value	Linear	Quadratic	Cubic
Immune Organs											
Bursa	0.12	0.11	0.12	0.09	0.09	0.12	0.01	0.691	0.879	0.651	0.430
Spleen	0.07	0.12	0.10	0.10	0.10	0.08	0.01	0.078	0.217	0.091	0.125
Thymus	0.16	0.16	0.15	0.16	0.12	0.18	0.02	0.609	0.540	0.498	0.580
Antibody Titer											
^1^ ND (Log_2_)	9.0 ^ab^	8.7 ^b^	8.8 ^b^	9.3 ^a^	9.1 ^ab^	9.4 ^a^	0.03	0.021	0.078	0.049	0.148
^2^ IBV	1503 ^b^	1573 ^ab^	1564 ^ab^	1600 ^a^	1553 ^ab^	1597 ^a^	0.02	0.032	0.012	0.049	0.089

^1^ ND: Newcastle disease, ^2^ IBV: infectious bronchitis virus, SEM: standard error of means based on 8 replicates, ^a,b^ Means within a row with (^a^, ^ab^, ^b^) superscripts differ significantly (*p* < 0.05).

## Data Availability

The data will be provided by the corresponding author.
